# Associations between dietary diversity score and migraine headaches: the results from a cross-sectional study

**DOI:** 10.3389/fnut.2023.1206278

**Published:** 2023-08-17

**Authors:** Shahnaz Amani Tirani, Gholamreza Askari, Fariborz Khorvash, Atefeh As’habi, Arman Arab

**Affiliations:** ^1^Department of Community Nutrition, School of Nutrition and Food Sciences, Isfahan University of Medical Sciences, Isfahan, Iran; ^2^Isfahan Neurosciences Research Center, Alzahra Hospital, Isfahan University of Medical Sciences, Isfahan, Iran; ^3^Food Safety Research Center (salt), Semnan University of Medical Sciences, Semnan, Iran

**Keywords:** migraine, headache, dietary diversity score, nutrition, Iran

## Abstract

**Aims:**

There is limited evidence on the link between diet quality and migraine headaches. The present study aimed to evaluate the association between dietary diversity score (DDS), as a good representative of overall diet quality, and clinical features of migraine headaches.

**Methods:**

In total, 262 subjects (224 females and 34 males), aged 20 to 50  years old were included in the present cross-sectional study. The migraine headache was diagnosed according to the third edition of the International Classification of Headache Disorders (ICHD-3). Clinical features of migraine headaches including frequency, severity, and duration of migraine headaches, headache impact test-6 (HIT-6), and serum levels of nitric oxide (NO) were assessed by standard procedures. The dietary intake of participants has been assessed by a validated 168-item food frequency questionnaire (FFQ) and used to calculate DDS. The association between DDS and clinical variables of migraine headaches was investigated using multiple linear regression analysis, and the beta (*β*) estimates with 95% confidence intervals (CIs) were reported.

**Results:**

A significant inverse association was found between DDS and headache frequency (*β* = −2.19, 95% CI: −4.25, −0.14) and serum levels of NO (*β* = −6.60, 95% CI: −12.58, −0.34), when comparing patients in the third tertile of DDS to those in the first tertile. The association remained significant and became stronger after adjustment for confounders for both outcomes of headache frequency (*β* = −3.36, 95% CI: −5.88, −0.84) and serum levels of NO (*β* = −9.86, 95% CI: −18.17, −1.55). However, no significant association was found between DDS with HIT-6 score, migraine headache duration, and severity.

**Conclusion:**

The present study demonstrates that higher dietary diversity is correlated with lower migraine frequency and serum levels of NO.

## Introduction

Migraine is a prevalent primary headache disorder, manifested by recurrent episodes of unilateral headache often associated with nausea, vomiting, photophobia, and/or phonophobia. Based on the study of Global Burden of Diseases, Injuries, and Risk Factors (GBD) 2019, migraine affects more than 10% of the general population and has been ranked as the second cause of years lived with disability (YLDs), especially in the working-age population (1, 2). Furthermore, migraine co-exists with several diseases such as depression, anxiety, chronic neck, and low back pain (3–7). As a result, migraine imposes a substantial burden on society because of direct healthcare costs as well as indirect costs due to work absenteeism and productivity loss (8, 9).

Given the pathophysiological complexity of migraine, limited progress has been made in disease management up to now (10). Nitric oxide (NO) seems to play a principal role in the pathogenesis of migraine (11). In addition, the trigeminovascular system inflammation and the dilation of cerebral vessels occurring during the episodes of migraine headaches have been attributed to the role of NO in the pathogenesis of migraine (12). However, the exact pathophysiology of migraine is not well understood and therefore further attempts are needed to investigate pathogenesis-related determinants of migraine.

In recent years, non-pharmacological approaches for migraine management have received considerable attention due to the low efficacy and tolerability as well as side effects of medications used in the treatment of migraine headaches (13, 14). The pathophysiology of migraine is complex and still not well understood, but the available data suggest that various aspects of diet such as diet-related triggers and dietary patterns can influence migraine attacks (15–18). According to previous studies, 12–60% of patients with migraine reported foods as triggers of headache attacks with many citing more than one dietary trigger (19). Specific foods such as caffeine, chocolate, milk, cheese, and alcoholic beverages have been reported as common migraine triggers (20). Additionally, dietary interventions such as low-fat diet (21–23), low glycemic index diet (24), elimination diet (25–27), ketogenic diet (28, 29), and dietary approach to stop hypertension (DASH) diet (30) have shown promising therapeutic benefits for migraine. Nevertheless, to routinely use dietary approaches in the management of migraine, further research on the association between diet and clinical characteristics of migraine headaches is needed.

The relationship between migraine headaches and diet quality has been demonstrated by some previous reports (31–33). For example, the results of the study conducted by Evans et al. in the framework of the National Health and Nutrition Examination Study (NHANES) 1999–2004 revealed that diet quality, measured by healthy eating index (HEI)-2005, in normal-weight females with migraine was significantly lower than those without migraine (31). The results of a cross-sectional study on Iranian women with migraine also showed that the quality of diet, assessed by HEI-2015, in women with chronic migraine was lower compared to those with episodic migraine (32). Bakirhan et al. in a study on patients with episodic migraine reported an inverse relationship between the severity of migraine attacks and HEI-2010 (33). More recently, Khorsha et al. revealed that dietary diversity, a surrogate measure of overall diet quality and nutrition adequacy, was inversely associated with headache frequency, pain severity, and migraine disability (34).

Therefore, we postulated that possibly dietary diversity is correlated with clinical characteristics of migraine through the mediating role of NO. Thus, the present study was undertaken to investigate the association between dietary diversity score (DDS) with clinical outcomes of migraine (i.e., frequency, duration, and severity of migraine headaches), migraine-related disability, and serum levels of NO in a sample of Iranian individuals.

## Methods

### Study design and participants

The current cross-sectional study was performed from August 2019 to June 2020 among 262 patients with migraine. A convenience sampling method was used to recruit patients from two referral neurology clinics of Imam Musa Sadr and Khorshid both affiliated with Isfahan University of Medical Sciences (IUMS), Isfahan, Iran. The required sample size was estimated to be 265, based on a previous study on Iranian patients with migraine (35), and considering type I error of 0.05, a power of 80%, a confidence interval of 95%, a correlation coefficient of 0.25, and drop-out rate of 10%. The study protocol was conducted according to the ethical guidelines of the 1975 Declaration of Helsinki and approved by the Ethics Committee of IUMS (IR.MUI.RESEARCH.REC.1398.352). Written informed consent was obtained from all participants before the study initiation. Patients were considered for inclusion in the study if they were between 20 to 50 years of age; were diagnosed with migraine according to the third edition of the International Classification of Headache Disorders (ICHD-3) (7), and had a body mass index (BMI) within 18.5–29.9 kg/m^2^. Patients with a history of diabetes, hypertension, cancer, other neurological disorders, cardiovascular, hepatic, thyroid, and renal diseases, as well as patients who had used dietary and herbal supplements (i.e., magnesium, coenzyme Q10, riboflavin, or feverfew), were not included in the study. Patients with a reported daily energy intake of lower than 800 kcal (3347 KJ/day) or higher than 4200 kcal (17537 KJ/day) were also excluded from analyses (36).

### Dietary intake assessment

A Persian-validated and reliable interviewer-administered 168-item semi-quantitative food frequency questionnaire (FFQ) was applied to assess dietary intakes (37). Patients were asked to report the consumption frequency of each food item during the preceding year on a daily, weekly, or monthly basis. The portion size of consumed food was converted to grams using household measures. The content of energy and nutrients of each food or beverage was computed by Nutritionist 4 software (First Databank, Hearst Corp., San Bruno, CA, United States).

### DDS calculation

Five dietary groups were used to calculate DDS, including dairy products, grains, vegetables, fruits, and meats based on the United States Department of Agriculture (USDA) food guide pyramid (38). Then, the main groups were divided into 23 subgroups according to Kant et al. (39) as follows: (a) the dairy group contained three subgroups of milk, yogurt, and cheese, (b) the grain group contained seven subgroups of refined flour, biscuits, whole grain cereals, pasta, rice, whole grain bread, and white bread, (c) the vegetable group contained seven subgroups of cruciferous vegetables, yellow vegetables, other vegetables, legumes, starchy vegetables, tomatoes, and potatoes, (d) the fruits group contained two subgroups of fruit and fruit juice as well as berries and citrus, and (e) the meat group was divided into four subgroups of red meat, poultry, fish, and egg. To be considered a consumer of any food group, each individual must have consumed at least half a serving of that food group in 1 day. To calculate the total score of DDS, the scores obtained from all subgroups were summed and divided by the number of subgroups in each group and then multiplied by two. For example, the subgroup score of a respondent who consumed at least half a serving of two of seven subgroups of grains in a day would be (2/7) x2 = 0.57. Thus, each food group DDS and total DDS ranged from 0 to 2 and 0 to 10, respectively.

### Migraine headaches assessment

Migraine-related disability was assessed by the short-form headache impact test (HIT-6) at the first visit (40). The questionnaire comprises 6 items, and all are answered using 5 response options: never (6 scores), rarely (8 scores), sometimes (10 scores), very often (11 scores), and always (13 scores). HIT-6 total scores ranged from 36 to 78, with higher scores indicating greater impact as classified into the following categories: none (36-49), moderate (50-55), substantial (56-59), and severe (≥ 60). Furthermore, patients received a 30-day headache diary as well as verbal and written instructions on how to fill out the diary during the forthcoming month to obtain clinical outcomes of migraine headaches including time of attack onset, duration, and severity of attacks. The severity of headaches was assessed by the visual analog scale (VAS). On this 10-point scale, 0 indicates no pain, while 10 indicates very severe pain (41). Patients were also asked to record the number of attacks (frequency) and mean duration of headache attacks by day (duration) per month in the headache diary.

### Other variables

The basic data on the patient’s sex, age, smoking, marital status, number of family members, family history of migraine, time since migraine diagnosis, migraine type (chronic or episodic), migraine characteristics (migraine with or without aura), and medications were collected using a general questionnaire at the first visit. An Iranian-validated version of the International Physical Activity Questionnaire (IPAQ) was used to measure patients’ physical activity status (42). The questionnaire assesses the average daily time spent on vigorous-intensity activity, moderate-intensity activity, walking, and sitting during the last 7 days. Metabolic Equivalent hours per day (MET-h/d) were estimated based on IPAQ data (43).

Blood pressure was measured in the sitting position twice after 10 min of rest using a mercury sphygmomanometer (Riester, Germany). Anthropometric variables were measured based on standard protocols by a trained dietitian. Weight was measured to the nearest 100 g with light clothes and without shoes using a digital scale (Omron BF511, Omron Corp., Kyoto, Japan). An upstretched tape meter was used to measure height to the nearest 1 mm without shoes. The BMI was computed using weight (kg) divided by height squared (m^2^).

Five milliliters of venous blood were taken from patients after 8 to 12 h of fasting. After the centrifuge at 3500 rpm, the serum was separated and kept at −80°C for biochemical tests. Serum NO was measured using the Griess method with available commercial kits (Kiazist Life Sciences, Iran).

### Statistical analysis

Data were analyzed by statistical package for the social sciences (SPSS) version 26 (IBM Corp, Armonk, NY, United States) and a *p*-value <0.05 was considered statistically significant. Quantitative and qualitative variables were expressed as mean [95% confidence interval (CI)], and number (percentage), respectively. The difference of quantitative variables across tertiles of DDS was examined via one-way analysis of variance (ANOVA). The distribution of categorical variables across tertiles of DDS was assessed using the Chi-square test. The association between DDS and headache duration, frequency, severity, migraine-related disability, and serum levels of NO were investigated using multiple linear regression analysis, and the beta (*β*) estimates with 95% CI were reported. In the first model, age, sex, and total energy intake were adjusted. Further adjustment was made for marital status, smoking status, migraine type, family history of migraine, mean arterial pressure, medication, and physical activity in the second model. The last model was additionally controlled for BMI.

## Results

**Figure 1:**
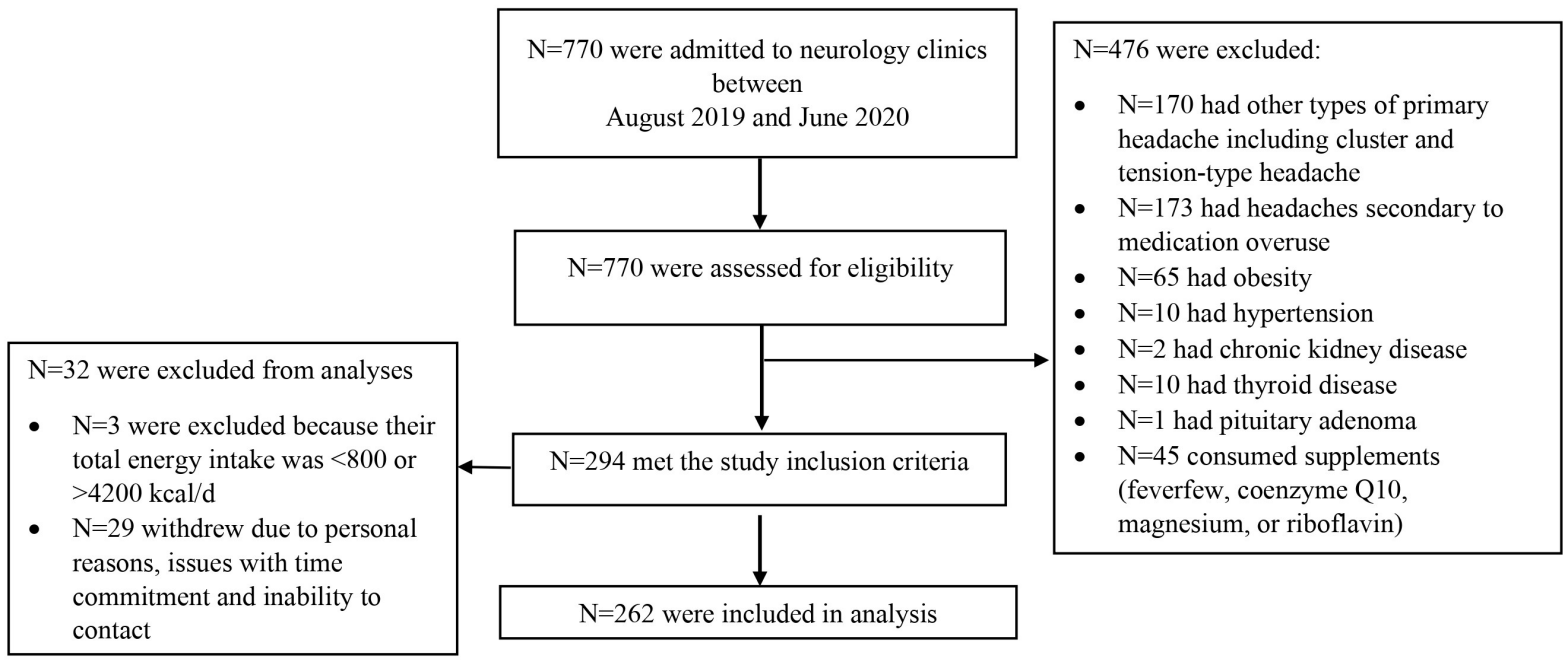
Flow diagram of the selection process of study participants.

In total, 770 patients were screened for possible eligibility to be included in the current study, of whom 265 met the inclusion criteria and three were excluded because their reported energy intake was outside the normal range of 800–4200 kcal/day. Therefore, the final analysis was performed on 262 patients comprising 224 females and 34 males (Figure 1). Patients had a mean (standard deviation) age of 36.10 (8.62) years, a BMI of 25.55 (3.44) kg/m^2^, a headache frequency of 7.80 (7.00) attacks/month, a headache duration of 0.96 (0.82) day/month, headache severity of 7.77 (1.78), and HIT-6 scores of 62.72 (7.17).

The basic socio-demographic and clinical outcomes of migraine patients according to DDS tertiles are shown in Table 1. Subjects with the highest values of DDS were less likely to be female (*p* = 0.032) and triptan consumers (*p* = 0.025). The number of patients with a history of migraine in the first-degree relatives was significantly higher in the highest DDS tertile (*p* = 0.018). Likewise, the frequency of migraine with aura was higher among patients in the second tertile of DDS (*p* = 0.021). No significant difference was observed regarding other basic characteristics of migraine patients across categories of DDS (all *p* > 0.05).

**Table 1:** Characteristics of the study population stratified by tertiles (T) of dietary diversity score (DDS).

Variables	Tertiles of DDS			*p value*
	T1 [<4.08]	T2 [4.08 to 6.10]	T3 [>6.10]	
*N*	87	88	87	
Age (y)	37.28 [35.40, 39.17]	36.55 [34.76, 38.34]	34.45 [32.66, 36.25]	0.080
Female	79 (90.8)	76 (86.4)	69 (79.3)	0.032
Married	73 (83.9)	73 (83.0)	66 (75.9)	0.178
Current smoker	4 (4.6)	6 (6.8)	5 (5.7)	0.745
Number of family members (n)	3.29 [3.08, 3.51]	3.43 [3.22, 3.63]	3.51 [3.30, 3.72]	0.342
Weight (kg)	67.31 [65.17, 69.45]	66.28 [64.06, 68.50]	69.88 [67.43, 72.33]	0.074
Height (cm)	161.09 [159.52, 162.66]	162.53 [160.99, 164.06]	164.94 [163.15, 166.73]	0.004
BMI (kg/m^2^)	25.93 [25.21, 26.66]	25.04 [24.36, 25.72]	25.67 [24.89, 26.45]	0.211
Physical activity (MET/h/d)	5.14 [2.96, 7.32]	10.11 [4.06, 16.16]	11.36 [7.37, 15.36]	0.111
SBP (mmHg)	113.50 [111.73, 115.27]	111.95 [109.79, 114.10]	112.52 [110.27, 114.78]	0.568
DBP (mmHg)	76.16 [74.68, 77.63]	74.67 [72.94, 76.39]	75.63 [74.22, 77.04]	0.389
MAP (mmHg)	88.60 [87.19, 90.02]	87.09 [85.39, 88.79]	87.93 [86.35, 89.50]	0.399
Migraine in first-degree relatives	50 (57.5)	52 (59.1)	65 (74.7)	0.018
Time since migraine diagnosis (y)	8.44 [6.40, 10.49]	7.24 [5.42, 9.05]	6.31 [4.65, 7.97]	0.268
Episodic migraine	72 (82.8)	71 (80.7)	73 (83.9)	0.842
Migraine with aura	26 (29.9)	42 (47.7)	41 (47.1)	0.021
Frequency (attacks per month)	8.56 [6.89, 10.23]	8.47 [6.82, 10.12]	6.36 [5.34, 7.38]	0.064
Duration (day/attack)	0.89 [0.72, 1.06]	0.97 [0.78, 1.16]	1.02 [0.85, 1.20]	0.567
Severity (visual analog scale)	7.85 [7.45, 8.24]	7.70 [7.30, 8.10]	7.78 [7.43, 8.12]	0.864
HIT-6 (score)	63.43 [61.85, 65.01]	62.25 [60.85, 63.64]	62.48 [60.87, 64.08]	0.513
Nitric oxide (nmol/mL)	36.72 [31.76, 41.69]	35.52 [30.82, 40.21]	30.12 [26.36, 33.88]	0.093
Taking beta-blockers	31 (35.6)	47 (53.4)	30 (34.5)	0.878
Taking topiramate	3 (3.4)	3 (3.4)	7 (8.0)	0.163
Taking TCAs	39 (44.8)	38 (43.2)	45 (51.7)	0.363
Taking TeCAs	4 (4.6)	2 (2.3)	2 (2.3)	0.379
Taking SNRIs	3 (3.4)	7 (8.0)	4 (4.6)	0.737
Taking sodium valproate	13 (14.9)	12 (13.6)	8 (9.2)	0.254
Taking triptans	20 (23.0)	14 (15.9)	9 (10.3)	0.025
Taking gabapentin	16 (18.4)	13 (14.8)	14 (16.1)	0.683
Taking benzodiazepine	2 (2.3)	8 (9.1)	3 (3.4)	0.728

Data are presented as mean [95% confidence interval] or number (% within tertiles of DDS). *p*-value obtained from chi-square analysis for categorical variables and one-way analysis of variance (ANOVA) for continuous variables. *p* < 0.05 was considered statistically significant. BMI, body mass index; SBP, systolic blood pressure; DBP, diastolic blood pressure; MAP, mean arterial pressure; TCA, tricyclic antidepressants; TeCA, tetracyclic antidepressant; SNRI, serotonin-norepinephrine reuptake inhibitor; HIT, headache impact test; DDS, dietary diversity score.

Macronutrients and food group intake of the study population across DDS tertiles are presented in Table 2. Patients in the highest tertile of DDS had a significantly higher intake of total energy, protein, pasta, refined flour, tomato, starchy vegetables, yellow vegetables, cruciferous vegetables, fruit and fruit juice, berries and citrus, and milk, as well as a lower intake of fat and rice (all *p* < 0.05).

**Table 2:** Selected food groups and macronutrient intake of participants across tertiles (T) of dietary diversity score (DDS).

Variables	Tertiles of DDS			*p value*
	T1 [<4.08]	T2 [4.08 to 6.10]	T3 [>6.10]	
Energy (Kcal/day)^a^	2270.48 [2157.80, 2383.16]	2550.21 [2447.33, 2653.10]	3114.84 [2981.72, 3247.97]	<0.001
Protein (g/d)	69.12 [65.50, 72.75]	70.46 [67.31, 73.61]	79.83 [75.23, 84.43]	<0.001
Fat (g/d)	115.45 [110.86, 120.04]	109.47 [105.39, 113.56]	107.86 [103.27, 112.45]	0.042
Carbohydrate (g/d)	352.14 [342.33, 361.94]	367.01 [357.81, 376.22]	365.03 [353.80, 376.25]	0.085
White breads (g/d)	67.71 [60.81, 74.62]	66.27 [60.72, 71.81]	69.21 [59.90, 78.53]	0.857
Whole-grain breads (g/d)	6.80 [3.12, 10.47]	9.73 [4.15, 15.32]	7.28 [1.86, 12.71]	0.677
Rice (g/d)	351.60 [319.52, 383.68]	345.71 [308.25, 383.18]	272.96 [233.30, 312.61]	0.004
Pasta (g/d)	11.48 [9.60, 13.36]	13.36 [10.93, 15.78]	17.50 [13.08, 21.92]	0.023
Whole-grain cereals (g/d)	13.59 [5.16, 22.02]	13.95 [8.12, 19.78]	15.31 [7.00, 23.63]	0.945
Biscuits (g/d)	6.76 [5.24, 8.28]	5.74 [4.46, 7.01]	8.35 [4.37, 12.33]	0.368
Refined flour (g/d)	5.52 [3.88, 7.16]	6.72 [5.09, 8.34]	11.54 [8.09, 14.98]	0.001
Potato (g/d)	25.09 [18.08, 32.10]	29.59 [22.49, 36.69]	24.06 [19.77, 28.36]	0.415
Tomato (g/d)	72.56 [57.04, 88.07]	86.02 [68.77, 103.27]	110.08 [92.17, 127.98]	0.007
Starchy vegetables (g/d)	2.15 [1.21, 3.10]	3.02 [2.11, 3.92]	4.90 [2.85, 6.95]	0.022
Legumes (g/d)	41.71 [32.74, 50.67]	42.23 [32.82, 51.64]	48.47 [38.00, 58.94]	0.543
Yellow vegetables (g/d)	14.58 [10.95, 18.21]	16.82 [11.77, 21.88]	25.24 [18.26, 32.22]	0.015
Cruciferous vegetables (g/d)	91.88 [77.81, 105.96]	124.42 [102.39, 146.45]	161.88 [130.61, 193.16]	<0.001
Other vegetables (g/d)	49.65 [43.22, 56.07]	54.93 [47.61, 62.26]	62.32 [53.70, 70.94]	0.059
Fruit and fruit juice	346.33 [311.24, 381.41]	428.50 [381.42, 475.58]	513.95 [459.64, 568.25]	<0.001
Berries and citrus	77.81 [67.01, 88.61]	96.34 [83.92, 108.76]	140.97 [123.77, 158.17]	< 0.001
Milk (g/d)	99.86 [70.80, 128.92]	80.88 [57.18, 104.59]	146.78 [100.61, 192.94]	0.024
Yogurt (g/d)	251.93 [191.33, 312.54]	294.83 [226.73, 362.92]	297.02 [236.98, 357.05]	0.525
Cheese (g/d)	18.04 [14.54, 21.54]	22.66 [17.29, 28.02]	24.47 [20.11, 28.84]	0.113
Red meat (g/d)	34.11 [25.81, 42.41]	30.39 [24.37, 36.41]	42.75 [32.73, 52.76]	0.101
Poultry (g/d)	13.77 [11.67, 15.88]	16.99 [13.67, 20.31]	18.20 [9.44, 26.97]	0.524
Fish (g/d)	3.70 [2.75, 4.64]	3.97 [2.72, 5.23]	5.34 [3.82, 6.86]	0.148
Egg (g/d)	20.88 [16.85, 24.92]	21.20 [17.91, 24.48]	26.80 [20.72, 32.89]	0.132

Data are presented as mean [95% confidence interval] and obtained from analysis of variance (ANOVA). *p* < 0.05 was considered statistically significant. DDS, dietary diversity score. ^a^Selected food groups and nutrients were adjusted for total energy intake.

Crude and multivariate-adjusted estimates and 95% CIs for the HIT-6 score, serum level of NO, frequency, duration, and severity of migraine headaches among DDS tertiles are summarized in Table 3. The frequency of headache attacks decreased in patients in the last DDS tertile compared to those in the first tertile (*β* = −2.19, 95% CI: −4.2, −0.14, P_trend_ = 0.037) according to the crude model. The association remained significant after adjustment for age, sex, and total energy intake (*β* = −3.35, 95% CI: −5.86, −0.85, P_trend_ = 0.010); marital status, smoking status, migraine type, family history of migraine, mean arterial pressure, medication, and physical activity (*β* = −3.38, 95% CI: −5.90, −0.86, P_trend_ = 0.009); and BMI (*β* = −3.36, 95% CI: −5.88, −0.84, P_trend_ = 0.010). Additionally, an inverse association was found between NO and DDS, when comparing patients in the third tertile of DDS to those in the first tertile (*β* = −6.60, 95% CI: −12.58, −0.34, P_trend_ = 0.039). This association remained significant after adjustment for age, sex, and total energy intake (*β* = −8.54, 95% CI: −16.70, −0.37, P_trend_ = 0.042); marital status, smoking status, migraine type, family history of migraine, mean arterial pressure, medication, and physical activity (*β* = −9.67, 95% CI: −17.98, −0.1.35, P_trend_ = 0.024); and BMI (*β* = −9.86, 95% CI: −18.17, −1.55, P_trend_ = 0.021). No significant association was found between DDS and HIT-6 score, severity, and duration of migraine attacks in the crude and adjusted models.

**Table 3:** Beta (*β*) and 95% confidence interval for clinical outcomes of migraine headaches according to tertiles (T) of dietary diversity score (DDS).

	Tertiles of DDS			
	T1 [<4.08]	T2 [4.08 to 6.10]	T3 [>6.10]	*p* trend
Frequency				
Crude	Ref	−0.08 (−2.13, 1.96)	−2.19 (−4.25, −0.14)	0.037
Model 1	Ref	−0.88 (−3.04, 1.27)	−3.35 (−5.86, −0.85)	0.010
Model 2	Ref	−1.19 (−3.34, 0.96)	−3.38 (−5.90, −0.86)	0.009
Model 3	Ref	−1.15 (−3.32, 1.01)	−3.36 (−5.88, −0.84)	0.010
Duration				
Crude	Ref	0.07 (−0.16, 0.32)	0.13 (−0.11, 0.37)	0.286
Model 1	Ref	0.08 (−0.19, 0.36)	0.26 (−0.05, 0.58)	0.103
Model 2	Ref	0.097 (−0.17, 0.37)	0.25 (−0.07, 0.57)	0.130
Model 3	Ref	0.08 (−0.18, 0.36)	0.24 (−0.07, 0.56)	0.138
Severity				
Crude	Ref	−0.14 (−0.67, 0.38)	−0.06 (−0.59, 0.45)	0.798
Model 1	Ref	−0.31 (−0.87, 0.25)	0.08 (−0.56, 0.74)	0.864
Model 2	Ref	−0.29 (−0.85, 0.26)	0.15 (−0.49, 0.80)	0.721
Model 3	Ref	−0.27 (−0.83, 0.28)	0.16 (−0.48, 0.81)	0.685
HIT-6				
Crude	Ref	−1.18 (−3.30, 0.93)	−0.95 (−3.07, 1.16)	0.379
Model 1	Ref	−0.93 (−3.28, 1.41)	0.24 (−2.47, 2.97)	0.911
Model 2	Ref	−0.94 (−3.30, 1.40)	0.19 (−2.55, 2.94)	0.944
Model 3	Ref	−1.13 (−3.48, 1.22)	0.08 (−2.65, 2.82)	0.995
Nitric oxide				
Crude	Ref	−1.20 (−7.44, 5.03)	−6.60 (−12.85, −0.34)	0.039
Model 1	Ref	−3.08 (−10.13, 3.95)	−8.54 (−16.70, −0.37)	0.042
Model 2	Ref	−3.30 (−10.42, 3.81)	−9.67 (−17.98, −1.35)	0.024
Model 3	Ref	−3.64 (−10.78, 3.50)	−9.86 (−18.17, −1.55)	0.021

Data are presented as *β* (95% confidence interval) and obtained from linear regression. Crude: Unadjusted. Model 1: Adjusted for age, sex, and total energy intake. Model 2: Model 1+ marital status, smoking status, migraine type, family history, mean arterial pressure, medication, and physical activity. Model 3: Model 2 + body mass index. *p* < 0.05 was considered statistically significant. HIT, headache impact test; DDS, dietary diversity score.

## Discussion

DDS is known as a key indicator of diet quality, and several past researches have investigated the relationship between this index and health outcomes. According to the results of these studies, dietary diversity is associated with reduced risk of depression, metabolic syndrome, abdominal obesity, and cardiovascular disease among the Iranian population (44–47). The current study showed an inverse significant association between diet quality, indicated by DDS, and some clinical outcomes of a migraine headache including frequency of migraine attacks and serum levels of NO even after adjustment for potential confounders. To the best of our knowledge, the present study is among the first to demonstrate the relationship between DDS and migraine headaches. Khorsha et al. previously expressed a significant inverse association between DDS with migraine-related disability, pain severity, and headache frequency in 256 Iranian women aged 18 to 50 years old. However, they did not present the consumption of various food groups based on DDS. In addition, the confounding role of some factors such as total energy intake and migraine type was neglected in their study (34).

The quality of diet in patients with migraine is low and probably affected by factors such as type of migraine, psychiatric comorbidities, obesity, and socioeconomic level (48). We found a link between diet quality with headache frequency and serum levels of NO which may be interpreted as a beneficial effect of DDS on migraine outcomes. However, it should be taken into account that this is only an association and bidirectionality may exist. Therefore, it is possible that those with a higher frequency of migraine headaches omit more food triggers and also have lower diet quality owing to nausea and vomiting accompanied by a migraine headache (49, 50).

Previous research has indicated that consuming a diverse food, particularly consuming different types of plant foods, improves microbial profile (51, 52). Evidence provided in recent years by human and animal studies has implied a relationship between the gut microbiome and migraine headaches (53, 54). Therefore, it is assumed that one of the mechanisms through which consuming a more diverse diet can improve migraine headaches is the effect on the gut microbiota and brain-gut axis. There is also increasing evidence that oxidative stress plays an important role in migraine pathogenesis (55–60). Thus, consuming a diverse diet mainly with greater amounts of fruits and vegetables are likely to improve migraine symptoms by modulating oxidative stress through receiving various antioxidants. In addition, higher DDS reflects the consumption of adequate micronutrients from a variety of foods which may improve migraine headaches. For example, an inverse association has been detected between dietary intake of some minerals such as magnesium (61, 62), calcium (62), iron (63), and zinc (64) with migraine. There are also some reports about the link between dietary intake of some vitamins such as folate (65), thiamin (66), and riboflavin (67) with migraine headaches.

We also assume that a significantly lower total fat intake in patients in the third tertile of DDS may provide some explanation for an inverse association between DDS and migraine headaches frequency. The efficacious role of a low-fat diet on migraine prophylaxis has been known by several interventional studies. Consuming a low-fat diet for at least 12 weeks has been associated with a significant reduction in headache frequency, severity, and duration (21–23). Some past studies have indicated the beneficial role of a high-fat, low-carbohydrate ketogenic diet in reducing migraine symptoms (28, 68, 69). However, it is important to note that the beneficial effects of a ketogenic diet on neurological diseases are contributed to ketone body production and blood sugar reduction (70).

A growing body of evidence implicated that NO plays an important role in the pathophysiology of migraine both as an independent factor and by interacting with a nitrergic cascade (71). Results of the present study indicated that there is an inverse association between dietary diversity score and serum levels of NO. Thus, it seems that dietary diversity enhancement may have a favorable effect on migraine headaches by targeting NO synthesis. Although, further large-scale prospective research is still essential to confirm this assumption and explore the causality.

Our study has some strengths. This is the second study investigating the association between DDS and migraine clinical features. Additionally, we presented, for the first time, information regarding the intake of macronutrients and food groups across various tertiles of DDS in migraine patients. Our study also has several limitations that should be acknowledged. Firstly, its cross-sectional design rules out definitive causal inferences between DDS and clinical outcomes of migraine. Therefore, prospective cohort research is warranted to establish a causal link between DDS and clinical outcomes of migraine headaches. Secondly, the generalizability of these findings is limited by a relatively small sample size and different dietary habits of our population compared to the population of other societies. Thirdly, despite applying FFQ as a validated and reliable questionnaire, the evaluation of dietary intakes was subject to recall and misclassification bias. Finally, although several covariates have been controlled in this study, uncontrolled confounders such as socioeconomic status, psychological, and environmental factors might affect our results.

## Conclusion

Our results indicated that the frequency of migraine attacks was reduced in patients in the third tertile of DDS compared to those in the first tertile, which could be related to reduced serum levels of NO. Further large-scale prospective studies are recommended to detect the association between the intake of diverse foods and migraine clinical outcomes.

## Data availability statement

The raw data supporting the conclusions of this article will be made available by the authors, without undue reservation.

## Ethics statement

The studies involving humans were approved by the study protocol was approved by the Ethics Committee of IUMS (IR.MUI.RESEARCH.REC.1398.352). The studies were conducted in accordance with the local legislation and institutional requirements. The participants provided their written informed consent to participate in this study.

## Author contributions

SA, GA, FK, AtA, and ArA contributed to the conception, design, data collection, data interpretation, and manuscript drafting. All authors contributed to the article and approved the submitted version.

## Conflict of interest

The authors declare that the research was conducted in the absence of any commercial or financial relationships that could be construed as a potential conflict of interest.

## Publisher’s note

All claims expressed in this article are solely those of the authors and do not necessarily represent those of their affiliated organizations, or those of the publisher, the editors and the reviewers. Any product that may be evaluated in this article, or claim that may be made by its manufacturer, is not guaranteed or endorsed by the publisher.

## Figures and Tables

**Table:** 

DDS	dietary diversity score
ICHD-3	International Classification of Headache Disorders
USDA	United States Department of Agriculture
HIT-6	headache impact test-6
NO	nitric oxide
GBD	Global Burden of Diseases, Injuries, and Risk Factors
YLDS	years lived with disability
FFQ	food frequency questionnaire
95% CI	95% confidence interval
BMI	body mass index
DASH	dietary approach to stop hypertension
IPAQ	International Physical Activity Questionnaire
MET-h/d	Metabolic Equivalent hours per day
VAS	visual analog scale
ANOVA	one-way analysis of variance
SPSS	statistical package for the social sciences
SE	standard error
SBP	Systolic Blood Pressure
DBP	Diastolic Blood Pressure
MAP	Mean Arterial Pressure
TCA	Tricyclic Antidepressants
TeCA	Tetracyclic Antidepressant
SNRI	Serotonin-Norepinephrine Reuptake Inhibitor
